# TGFBR1^*^6A and Int7G24A variants of transforming growth factor-*β* receptor 1 in Swedish familial and sporadic breast cancer

**DOI:** 10.1038/sj.bjc.6603961

**Published:** 2007-09-11

**Authors:** B Song, S Margolin, J Skoglund, X Zhou, J Rantala, S Picelli, B Werelius, A Lindblom

**Affiliations:** 1 Department of Molecular Medicine and Surgery, Karolinska Institutet, Stockholm 171 76, Sweden; 2 Department of Pathology, Dalian Medical University, Dalian 116027, China; 3 Department of Oncology, Karolinska University Hospital at Södersjukhuset, Stockholm 118 83, Sweden

**Keywords:** breast cancer, sporadic, familial, transforming growth factor-*β* receptor 1, polymorphism

## Abstract

Two common variants in transforming growth factor-*β* receptor 1 (*TGFBR1*), TGFBR1^*^6A and Int7G24A, A allele, have been shown to act as low-penetrance tumour susceptibility alleles in several common cancers, including breast cancer. We evaluated the *TGFBR1* 9A/6A and Int7G24A variant frequencies in two breast cancer cohorts; a population-based cohort of breast cancer with defined family history (*n*=459) and in breast cancer patients from a familial cancer clinic (*n*=340) and in 856 controls from the Stockholm region. The familial patients from both cohorts were further divided into high- and low-risk familial breast cancer based on pedigree analysis. There was no overall association with either variant and breast cancer risk. The TGFBR1^*^6A allelic frequency was, however, higher in low-risk familial breast cancer (0.138), compared to controls (0.106; *P*=0.04). No significant difference was found in the high-risk familial (0.102) or sporadic cases (0.109; *P*=0.83 and 0.83, respectively). TGFBR1^*^6A carrier status was further associated with a high-grade sporadic breast cancer (odds ratio: 2.27; 95% confidence interval: 1.01–5.11; *P*=0.049). These results indicate that the TGFBR1^*^6A variant may be associated with an increased risk of low-risk familial breast cancer and might be a marker for poorly differentiated breast cancer. The Int7G24A variant was not associated with breast cancer risk or clinical presentation of the disease including prognosis in our material.

Breast cancer is the most common female malignancy in the Western countries (Parkin *et al*, 1999), and in Sweden about 6600 new cases are diagnosed annually (The National Board of Health and Welfare, 2007). Although the majority of breast cancer cases are sporadic, approximately 25–30% exhibit a familial clustering and 5–10% do have a hereditary component (Newman *et al*, 1988; Margolin *et al*, 2006). Mutations in the known high-risk genes, *BRCA1*, *BRCA2*, *p53*, *ATM* and *PTEN*, account for less than 25% of the familial risk for breast cancer, and in the Stockholm region the frequency of mutations in *BRCA1* and *BRCA2* is less than 10% in families with three or more cases with breast cancer (Arver *et al*, 2001; Thompson and Easton, 2004). It is reasonable that the remaining breast cancer families carry mutations in as yet unidentified high-penetrance genes, or are due to the combined effects of a large number of low-penetrance genes acting in a multiplicative manner (Pharoah *et al*, 2002). The latter hypothesis may also partly explain the development of sporadic breast cancer.

Disrupted transforming growth factor-*β* (TGF-*β*) signalling is involved in the development of various types of cancer. As one of the key effectors of TGF-*β* signalling, TGF-*β* receptor 1 (TGFBR1) mediates the growth-inhibitory signals from TGF-*β* through a complex with TGFBR2. Growing epidemiological evidence indicates that common variants of the *TGF-β* pathway receptors that alter TGF-*β* signalling can modify cancer risk (Kaklamani and Pasche, 2004). Two common alleles in the *TGFBR1* gene, TGFBR1^*^6A and Int7G24A, A allele, which reside in exon 1 and intron 7, respectively, have been reported to act as low-penetrance tumour susceptibility alleles (Chen *et al*, 1999, 2004, 2006; Pasche *et al*, 1999, 2004; Baxter *et al*, 2002; Kaklamani *et al*, 2003; Zhang *et al*, 2003; Bian *et al*, 2005; Kaklamani and Pasche, 2005).

TGFBR1^*^6A has a deletion of three alanines within a stretch of nine alanines. Functional investigations have shown that TGFBR1^*^6A is an impaired mediator of TGF-*β* antiproliferative signals compared with intact *TGFBR1* (Chen *et al*, 1999; Pasche *et al*, 1999). Several studies including a meta-analysis of 12 studies have demonstrated that TGFBR1^*^6A acts as a low-penetrance tumour susceptibility allele in the development of colon, cervix, breast, and ovarian cancer as well as haematological malignancies (Chen *et al*, 1999; Pasche *et al*, 1999, 2004; Baxter *et al*, 2002; Kaklamani *et al*, 2003; Bian *et al*, 2005; Kaklamani and Pasche, 2005).

The Int7G24A variant causes a G → A transversion in the +24 position of the donor splice site in intron 7 of *TGFBR1*. Although the functional role of this variant is still to be elucidated, Int7G24A has been found to increase the risk of kidney and bladder cancer and homozygous carriers have more than 3-fold increased risk of developing non-small-cell lung cancer (NSCLC) (Zhang *et al*, 2003; Chen *et al*, 2004). A case–control study indicated that this intronic variant allele was significantly more prevalent in invasive and metastatic breast cancer cases and may represent a marker for breast cancer progression (Chen *et al*, 2006).

In the present study, we evaluated the frequencies of *TGFBR1* 9A/6A and Int7G24A variants in two breast cancer cohorts with a defined family history and controls from Stockholm, Sweden and also examined whether these variants were associated with tumour presentation and prognosis.

## MATERIALS AND METHODS

### Material

#### Familial risk cohort

Three hundred and forty familial cases collected at the Department of Clinical Genetics at Karolinska University Hospital, Stockholm were used for the study. These patients had either been referred due to a breast cancer diagnosis and a family history of breast cancer or had been included as part of a previous research project on familial breast cancer (Lindblom, 1993). All cases had proceeded through genetic counselling and those who met the current criteria for *BRCA1/2* screening had been screened negative (Arver *et al*, 2001). For these cases, there was no information on tumour characteristics or prognosis.

#### Population-based cohort

Patients with a surgically treated primary invasive breast cancer admitted to the Department of Oncology at Huddinge Hospital and Söder Hospital (covering the population of southern Stockholm of 850 000 people) from October 1998 to May 2000 were asked participate in a study on genetic risk factors from breast cancer (Margolin *et al*, 2004). Family history, age at diagnosis, hormone receptor status and histology of the tumour were obtained from all cases, and the median follow-up was 5 years. This cohort consists of 489 patients in total and 459 patients were used in this study due to logistic reasons. The samples had previously been screened for mutations in exon 11 of BRCA1 (11). Four cases with known *BRCA1* or *BRCA2* mutations were excluded from the study.

The familial cases were divided into two groups based on pedigree analysis. Families with at least two first- or second-degree relatives with breast cancer in addition to the proband, regardless of age, were classified as high-risk breast cancer families. In this group, a dominant mode of inheritance is suggested. Cases with one first- or second-degree relative with breast cancer were classified as low-risk familial breast cancer, and in this group the mode of inheritance is unclear. This classification is not ideal from a risk-estimation point of view since the age factor is omitted but may reflect monogenic *vs* polygenic susceptibility better than a more strict estimation of breast cancer risk.

The mean age in the cases from the Department of Clinical Genetics was 54 years (24–92 years). In the cases from the Department of Oncology, the mean age was 60 years (27–88 years), and there was no statistically significant difference between familial and sporadic cases (mean age 59 and 61 years, respectively).

As controls, we used DNA from 856 blood donors of mixed gender collected at Karolinska University Hospital, Stockholm, Sweden.

The ethical committee at the Karolinska Institute approved of the study.

### Methods

#### TGFBR1^*^6A genotyping

PCR amplification of exon 1 was performed with the following primers: Fwd-5′-FamGAGGCGAGGTTTGCTGGGGTGAGG-3′ and Rev-5′-CATGTTTGAGAAAGAGCAGGAGCG-3′. The reactions were performed using supplied protocol for GC-rich fragments (Invitrogen, Paisley, UK) in a total volume of 25 *μ*l containing 50 ng DNA and 1.25 U Platinum® *Taq* DNA polymerase (Invitrogen). The fluorescently labelled PCR products were then separated on an ABI 377 DNA Sequencer (Applied Biosystems, Foster City, CA, USA). The genotypes were analysed using the GENESCAN™ and GENOTYPER™ software (Applied Biosystems). A product size of 247 bp represented the wild-type *TGFBR1* (9A) allele, whereas a product size of 255 bp represented the TGFBR1^*^6A allele.

#### Int7G24A genotyping

Genotyping primers for the Int7G24A variant were Fwd-5′TGTCTGAAAGGAGGTTCATCC-3′ and Rev-5′-GAACAACTTCTGCTCATGACG-3′. The PCR was carried out in a total volume of 25 *μ*l containing 50 ng DNA and 0.625 U AmpliTaq Gold® DNA polymerase (Applied Biosystems). A total volume of 10 *μ*l PCR product was digested using 3 U of *Bsr*1 restriction enzyme in 1 × NEBuffer3 at 65°C for 8 h. The digested DNA products were then separated in a 2.5% agarose gel and visualised by ethidium bromide staining. *Bsr*1 cuts the wild-type Int7G24A sequence while the *Bsr*1 restriction site is eliminated by the variant allele. For all identified variant carriers and for approximately 25% of the wild-type samples, restriction enzyme digestion was replicated after a second independent PCR amplification to confirm the allele calling. The concordance rate was 100%.

### Statistical analysis

The allelic frequency was analysed by the *χ*^2^-test. Genotype distribution in breast cancer cases and controls was tested for the Hardy–Weinberg equilibrium, and the two variants were shown to be in the Hardy–Weinberg equilibrium in both cases and controls.

Genotype data were compared between the groups using univariate logistic regression analysis for binomial and ordinal responses. For the ordinal response variable grade, we used a model where each non-reference category was contrasted with the reference category (grade 1). Results are presented as odds ratios (ORs) with 95% test-based confidence intervals (CIs). No correction for multiple statistical testing was performed.

## RESULTS

TGFBR1^*^6A genotypes were scored for 95.6% (764 out of 799) and 100% (856 out of 856) of the cases and controls, respectively, corresponding to genotype results for 309 sporadic, 254 low-risk and 201 high-risk familial cases and 856 controls. The Int7G24A variant was successfully genotyped in 96.0% (767 out of 799) of the cases and 99.6% (853 out of 856) of the controls, with genotype results for 311 sporadic, 250 low-risk and 206 high-risk cases and 853 controls.

There was no overall association with TGFBR1 6A allele and breast cancer, neither in the familial risk cohort (*P*=0.17) nor in the population-based cohort (*P*=0.25) compared to controls (Table 1a).

The TGFBR1^*^6A variant was found to be associated with an increased risk for breast cancer in the low-risk familial breast cancer group (Table 1b). The TGFBR1^*^6A allelic frequency was 0.138 in the low-risk familial cases compared to 0.106 in the controls (*P*=0.04) corresponding to an OR for ^*^6A carriers of 1.3 (95% CI: 1.0–1.9). The OR for ^*^6A/^*^9A and ^*^6A/^*^6A genotypes was 1.3 (95% CI: 0.9–1.8) and 2.1 (95% CI: 0.8–6.0), respectively, in this group. There was no difference in the TGFBR1^*^6A frequency in sporadic or high-risk familial breast cancer compared to controls (Table 1b). In addition to the ^*^9A and ^*^6A alleles, the ^*^11A allele was detected in one sporadic breast cancer case, and ^*^5A, ^*^7A and ^*^10A were detected in the controls.

The genotype distribution and allelic frequency of Int7G24A in the breast cancer cases and controls are provided in Table 2a and 2b. There was no overall association with this variant and breast cancer in either cohort (*P*=0.25 and 0.20, respectively, Table 2a). The A-allele frequency was slightly more common among low-risk familial (0.208) or sporadic breast cancer group (0.203) than among controls (0.183); however, this difference was not statistically significant. The ORs for Int7G24A carriers, GA heterozygotes and AA homozygotes, in these two groups all exceeded 1. The A-allele frequency was lower in the high-risk familial breast cancer group (0.158) compared to the controls, corresponding to ORs of A-allele carriers, including both heterozygotes and homozygotes, lower than 1 (Table 2b).

For the majority of the sporadic breast cancer cases from the Department of Oncology at Huddinge and Södersjukhuset Hospital, information on tumour grade, stage, ER and PR status and recurrence was available. The frequency of TGFBR1^*^6A carriers among patients diagnosed with grade 3 tumours was higher than among patients diagnosed with grade 1 tumours (OR: 2.27; 95% CI: 1.01–5.11; *P*=0.049, Table 3). There was no association of TGFBR1^*^6A with other clinical parameters, including tumour stage, ER and PR status at diagnosis and recurrence. In addition, no association was found between Int7G24A carriers and any of the clinical parameters mentioned above (Table 3).

## DISCUSSION

The role of TGFBR1^*^6A variant in breast cancer is controversial (Baxter *et al*, 2002; Jin *et al*, 2004; Kaklamani and Pasche, 2005). Baxter *et al* (2002) investigated the TGFBR1^*^6A variant in 355 breast cancer cases and 248 controls. Their results indicated that the ^*^6A allelic frequency was significantly higher in breast cancer patients compared to the controls. This result was further supported by a meta-analysis from 2004 on TGFRB1^*^6A and cancer susceptibility where there was a significant association of the variant with breast cancer. The OR for breast cancer was 1.38 (95% CI: 1.14–1.67) for carriers of the 6A allele, this figure based on 1400 breast cancer cases from seven different studies, including the Baxter study (Pasche *et al*, 2004). However, in a study of eight variants including the ^*^6A variant in Finnish unselected and Polish familial breast cancer cases, there was no association neither of any of the variants nor of haplotypes (Jin *et al*, 2004).

There are several explanations for difficulties reaching conclusive results on variants in candidate low-penetrance genes such as TGFRB1^*^6A. Association studies on variants conferring modest risks require large sample sizes; the study size is, however, also depending on variant frequency. The study size can be reduced by selecting cases enriched for genetic susceptibly such as patients with family history or bilateral cases (Houlston and Peto, 2004). We therefore chose to analyse the genotype distribution and allelic frequency of TGFBR1^*^6A in two cohorts of patients with a well-defined family history and classified the patients in high- and low-risk family history and sporadic breast cancer. There was no overall association with the TGFRB1^*^6A variant and breast cancer in either of our breast cancer cohorts. Our results indicate that the TGFBR1^*^6A variant might confer an increased risk of breast cancer in families classified as having a low-risk familial history, even though there was only statistical significance on the allelic level, and not in the genotype distribution. This therefore needs to be confirmed. Breast cancer tumours in TGFBR1^*^6A carriers were also slightly more poorly differentiated, indicating that the TGFBR1^*^6A variant might be associated with tumour grade even though our material of grade III tumours (*n*=75), from the population-based cohort, is small and the association of borderline significance only. Moreover, this result needs to be confirmed in a larger material.

Studies on Int7G24A are rare. This variant has been reported to be associated with NSCLC, kidney and bladder cancer susceptibility (Zhang *et al*, 2003; Chen *et al*, 2004). Patients with invasive and metastatic breast cancer have been reported to have a higher frequency of Int7G24A, and this variant may represent a marker for breast cancer progression (Chen *et al*, 2006). No studies have to date been published on this variant in familial breast cancer. In the present study, we compared the genotype distribution and allelic frequency of Int7G24A in 206 high-risk and 250 low-risk familial breast cancer cases, 311 sporadic breast cancer cases and 853 controls. The frequency of the Int7G24A variant in low-risk familial and sporadic breast cancer cases was higher compared to the controls, the OR for Int7G24A carriers and GA homozygotes and AA homozygotes as well, but the difference did not reach statistical significance. The Int7G24A variant may have an increased risk for low-risk familial and sporadic breast cancer, and this needs to be confirmed by a larger sample size.

In the high-risk familial breast cancer group, we found the frequency of both *TGFBR1* variants to be lower than among the controls. The different results obtained from subgrouping the families into high and low risk support the hypothesis that different genetic models might operate in these two groups of patients.

The controls in this study were blood donors of mixed gender from the same region. It has been suggested that matching for gender may not be relevant unless the variant causes early gender-specific mortality (which would result in a deviation from the Hardy–Weinberg equilibrium) (Hemminki and Forsti, 2002). The two variants of the *TGFBR1* gene in our controls were shown to be in the Hardy–Weinberg equilibrium. Chen *et al* (2004) found the A-allele frequency in male and female controls to be similar (0.137 and 0.145, respectively). A possible age difference between cases and controls, where the latter would presumably have a lower mean age and thus not had time to develop a breast tumour, could cause a false-negative result (Pasche *et al*, 1999; Kaklamani and Pasche, 2005).

In conclusion, there was no overall association of the *TGFBR1* variants ^*^6A and Int7G24A and breast cancer in two breast cancer cohorts from the Stockholm region. The ^*^6A variant seems to confer an increased risk of low-risk familial breast cancer. The Int7G24A variant may be associated with the development of low-risk familial and sporadic breast cancer; however, this needs to be confirmed in a larger material. Furthermore, the ^*^6A variant might be linked to poorly differentiated breast cancer.

## Figures and Tables

**Table 1a:** TGFBR1^*^6A genotypes in controls and in two Stockholm breast cancer cohorts

	**Controls (*n*=856)^a^**	**Familial risk cohort (*n*=327)**		**Population-based cohort (*n*=437)**	
**TGFBR1** ^*^ **6A genotypes**	**No. of cases (%)**	**No. of cases (%)**	**OR (95% CI)**	**No. of cases (%)**	**OR (95% CI)**
9A/9A	682 (79.7)	250 (76.4)	1.0	348 (79.6)	1.0
6A/9A	160 (18.7)	72 (22.0)	1.2 (0.9–1.7)	80 (18.3)	1.0 (0.7–1.3)
6A/6A	10 (1.2)	5 (1.5)	1.4 (0.5–4.0)	8 (1.8)	1.6 (0.6–4.0)
6A/9A+6A/6A	170 (19.9)	77 (23.5)	1.2 (0.9–1.7)	88 (20.1)	1.0 (0.8–1.3)
6A-allele freq.	0.11	0.12		0.11	
*P*-value‡		0.17		0.25	

CI=confidence interval; OR=odds ratio.

a Including the rare allele genotypes such as 5A, 7A, 10A and 11A.

‡ *P*-value for 6A-allele frequency comparison using *χ*^2^-test.

**Table 1b:** TGFBR1^*^6A genotypes in controls, high-risk and low-risk familial and sporadic breast cancer cases

	**Controls (*n*=856)^a^**	**High-risk familial breast cancer (*n*=201)**		**Low-risk familial breast cancer (*n*=254)**		**Sporadic breast cancer (*n*=309)^a^**	
TGFBR1*6A genotypes	**No. of cases (%)**	**No. of cases (%)**	**OR (95% CI)**	**No. of cases (%)**	**OR (95% CI)**	**No. of cases (%)**	**OR (95% CI)**
9A/9A	682 (79.7)	162 (80.6)	1.0	190 (74.8)	1.0	246 (79.6)	1.0
6A/9A	160 (18.7)	37 (18.4)	1.0 (0.7–1.4)	58 (22.8)	1.3 (0.9–1.8)	57 (18.4)	1.0 (0.7–1.4)
6A/6A	10 (1.2)	2 (1.0)	0.8 (0.2–3.9)	6 (2.4)	2.1 (0.8–6.0)	5 (1.6)	1.4 (0.5–4.1)
6A/9A+6A/6A	170 (19.9)	39 (19.4)	1.0 (0.7–1.4)	64 (25.2)	1.3 (1.0–1.9)	62 (20.1)	1.0 (0.7–1.4)
6A-allele freq.	0.106	0.102		0.138		0.109	
*P*-value‡		0.83		0.04		0.83	

CI=confidence interval; OR=odds ratio.

a Including the rare allele genotypes such as 5A, 7A, 10A and 11A.

‡ *P*-value for 6A-allele frequency comparison using *χ*^2^-test.

**Table 2a:** Int7G24A genotypes in controls and in two Stockholm breast cancer cohorts

	**Controls (*n*=853)**	**Familial risk cohort (*n*=323)**		**Population-based cohort (*n*=444)**	
**Int7G24A genotypes**	**No. of cases (%)**	**No. of cases (%)**	**OR (95% CI)**	**No. of cases (%)**	**OR (95% CI)**
GG	559 (65.5)	222 (68.7)	1.0	278 (62.6)	1.0
GA	265 (31.1)	93 (28.8)	0.9 (0.7–1.2)	145 (32.7)	1.1 (0.9–1.4)
AA	29 (3.4)	8 (2.5)	0.7 (0.3–1.5)	21 (4.7)	1.5 (0.8–2.6)
GA+AA	294 (34.5)	101 (31.3)	0.9 (0.7–1.1)	166 (37.4)	1.1 (0.9–1.4)
A-allele freq.	0.183	0.169		0.211	
*P*-value‡		0.25		0.20	

CI=confidence interval; OR=odds ratio.

‡ *P*-value for A-allele frequency comparison using *χ*^2^-test.

**Table 2b:** Int7G24A genotypes in controls, high-risk and low-risk familial and sporadic breast cancer cases

	**Controls (*n*=853)**	**High-risk familial breast cancer (*n*=206)**		**Low-risk familial breast caner (*n*=250)**		**Sporadic breast cancer (*n*=311)**	
**Int7G24A genotypes**	**No. of cases (%)**	**No. of cases (%)**	**OR (95% CI)**	**No. of cases (%)**	**OR (95% CI)**	**No. of cases (%)**	**OR (95% CI)**
GG	559 (65.5)	147 (71.4)	1.0	157 (62.8)	1.0	197 (63.3)	1.0
GA	265 (31.1)	53 (25.7)	0.8 (0.5–1.1)	82 (32.8)	1.1 (0.8–1.5)	102 (32.8)	1.1 (0.8–1.4)
AA	29 (3.4)	6 (2.9)	0.8 (0.3–1.9)	11 (4.4)	1.3 (0.7–2.8)	12 (3.9)	1.17 (0.6–2.3)
GA+AA	294 (34.5)	59 (28.6)	0.8 (0.5–1.1)	93 (37.2)	1.1 (0.8–1.5)	114 (36.7)	1.10 (0.8–1.4)
A-allele freq.	0.183	0.158		0.208		0.203	
*P*-value‡		0.14		0.35		0.47	

CI=confidence interval; OR=odds ratio.

‡ *P*-value for A-allele frequency comparison using *χ*^2^-test.

**Table 3:** Association of TGFBR1^*^6A genotypes with clinical parameters

	TGFBR1*6A genotypes						**Int7G24A genotypes**					
	**Total cases^a^**	**Available cases**	**9A/9A**	**6A/9A+6A/6A**	**OR (95% CI)**	***P*-values**	**Total cases**	**Available cases**	**GG**	**GA+AA**	**OR (95% CI)**	***P*-values**
*Grade*	309	274^a^					311	275				
1^b^		71	60	11	1.0			71	45	26	1.0	
2		127	103	24	1.3 (0.6–2.8)	0.55		129	77	52	1.2 (0.6–2.1)	0.61
3		75	53	22	2.3 (1.0–5.1)	0.05		75	49	26	0.9 (0.5–1.8)	0.81

*Stage*	309	309^a^					311	311				
Stage I/II^b^		273	218	55	1.0			277	176	101	1.0	
Stage III/IV		35	28	7	1.0 (0.4–2.4)	0.98		34	22	12	0.9 (0.4–2.0)	0.89

*ER status*	309	293^a^					311	294				
Positive^b^		245	196	49	1.0			247	154	93	1.0	
Negative		47	37	10	1.1 (0.5–2.3)	0.84		47	31	16	0.9 (0.4–1.6)	0.64

*PR status*	309	261^a^					311	261				
Positive^b^		199	162	37	1.0			200	123	77	1.0	
Negative		61	44	17	1.7 (0.9–3.3)	0.12		61	40	21	0.8 (0.5–1.5)	0.57

Recurrence	309	298^a^					311	297				
No^b^		245	197	48	1.0			245	153	92	1.0	
Yes		52	43	9	0.9 (0.4–1.9)	0.70		52	35	17	0.8 (0.4–1.5)	0.51

CI=confidence interval; OR=odds ratio.

a Including the rare genotype 11A.

b Were used as the reference.
